# AIM2 Inflammasome-Mediated Pyroptosis in Enterovirus A71-Infected Neuronal Cells Restricts Viral Replication

**DOI:** 10.1038/s41598-017-05589-2

**Published:** 2017-07-19

**Authors:** Thinesshwary Yogarajah, Kien Chai Ong, David Perera, Kum Thong Wong

**Affiliations:** 1 Department of Pathology, Faculty of Medicine, University of Malaya, Kuala Lumpur, Malaysia; 2 Department of Biomedical Science, Faculty of Medicine, University of Malaya, Kuala Lumpur, Malaysia; 3 Institute of Health and Community Medicine, University Malaysia Sarawak, Sarawak, Malaysia

## Abstract

Encephalomyelitis is a well-known complication of hand, foot, and mouth disease (HFMD) due to Enterovirus 71 (EV71) infection. Viral RNA/antigens could be detected in the central nervous system (CNS) neurons in fatal encephalomyelitis but the mechanisms of neuronal cell death is not clearly understood. We investigated the role of absent in melanoma 2 (AIM2) inflammasome in neuronal cell death, and its relationship to viral replication. Our transcriptomic analysis, RT-qPCR, Western blot, immunofluorescence and flow cytometry studies consistently showed AIM2 gene up-regulation and protein expression in EV-A71-infected SK-N-SH cells. Downstream AIM2-induced genes, CARD16, caspase-1 and IL-1β were also up-regulated and caspase-1 was activated to form cleaved caspase-1 p20 subunits. As evidenced by 7-AAD positivity, pyroptosis was confirmed in infected cells. Overall, these findings have a strong correlation with decreases in viral titers, copy numbers and proteins, and reduced proportions of infected cells. AIM2 and viral antigens were detected by immunohistochemistry in infected neurons in inflamed areas of the CNS in EV-A71 encephalomyelitis. In infected AIM2-knockdown cells, AIM2 and related downstream gene expressions, and pyroptosis were suppressed, resulting in significantly increased virus infection. These results support the notion that AIM2 inflammasome-mediated pyroptosis is an important mechanism of neuronal cell death and it could play an important role in limiting EV-A71 replication.

## Introduction

Enterovirus A71 (EV-A71) is a human RNA virus that belongs to the species A group, *Enterovirus* genus and *Picornaviridae* family. The virion is about 30 nm and contains a single-stranded, positive-sense RNA genome of approximately 7.5 kb. EV-A71 causes sporadic and epidemic hand, foot and mouth disease (HFMD), a common infectious disease most frequently seen in young children aged 5 and below^1–3^. Since its initial isolation and identification in 1969^4^, numerous large outbreaks of HFMD have been reported worldwide^5–13^. EV-A71-associated HFMD is occasionally associated with central nervous system (CNS) complications, such as aseptic meningitis, acute flaccid paralysis and encephalomyelitis^14–19^. Based on autopsy findings in fatal cases of EV-A71 encephalomyelitis, it is clear that CNS neurons are the main viral targets since neuronal degeneration/necrosis and neuronophagia were readily observed. Moreover, viral antigens and RNA localized almost exclusively to these cells^20, 21^. Thus, viral-induced cell death or viral cytolysis in neurons plays a major role in neuropathogenesis^22, 23^.

Classically, neuronal cell death may result from apoptosis and necrosis^24^. Nonetheless, recent advances in understanding of cell death mechanisms suggest that apart from apoptosis, other complex mechanisms such as pyroptosis, autophagy and necroptosis may be involved in viral infection^25–28^. Even though both pyroptosis and necroptosis are programed cell death mechanisms and promote inflammation, these pathways differ in their initiators; pyroptosis is induced via inflammasomes and caspase-1 activation, while necroptosis involves receptor-interacting protein kinase 3^29^. Moreover, both mechanisms are distinct from autophagy that causes activation of microtubule-associated protein 1A/1B-light chain 3 and formation of autophagosomes. Studies have shown that EV-A71 infection can cause apoptosis in cell lines such as rhabdomyosarcoma, human neuroblastoma (SK-N-SH, SK-N-MC and SH-SY5Y) and human glioblastoma cells^30–34^. Specifically, protein expression of cleaved caspase-9 was shown in EV-A71-infected SK-N-SH cells indicating cells undergo apoptosis. On the other hand, in our previous study, we have been unable to demonstrate apoptosis in SK-N-SH cells; the evidence had suggested neuronal necrosis^35^. Moreover, apoptosis has also not been convincingly demonstrated in infected CNS neurons in fatal human EV-A71 encephalomyelitis, although neuronal necrosis by viral cytolysis were well documented^20, 36–38^.

We investigated the specific mechanisms, which may be involved in neuronal death induced by EV-A71 as this phenomenon remains under-investigated. In particular, we examined the role of pyroptosis, a recently described novel programmed cell death mechanism which is characterized by caspase 1 activation, DNA breakages without laddering, cell swelling, plasma membrane rupture and release of intracellular contents of pro-inflammatory cytokines^39, 40^. Pyroptosis was first characterized in *Salmonella* ^41^ and *Shigella* ^42^ infections and recently also described in adenovirus, encephalomyocarditis virus and rhinovirus infections in non-neuronal human cell lines^43–45^. As far as we are aware, pyroptosis in neurons has only been described in acute brain injuries such as stroke and trauma, and neurodegenerative diseases such as Alzheimer’s and Parkinson’s diseases^46–48^.

During pyroptosis, caspase-1 forms part of, and is activated by, a large supramolecular complex known as inflammasome that may also comprise other proteins such as “PYD and CARD Containing Domain” (PYCARD). Inflammasome assembly may be initiated by various protein activators including “absent in melanoma 2” (AIM2), “NOD-like-Receptor Protein” (NLRP) or “RNA Sensor Retinoic Acid-inducible Gene-I” (RIG-I)^49–51^. AIM2-activated inflammasome or AIM2 inflammasome is known to be generally triggered by cytosolic DNA from both bacteria (*Listeria monocytogenes*)^52^ and viral (murine cytomegalovirus^53^ and vaccinia virus)^54, 55^. RNA viruses that trigger AIM2 inflammasome includes Chikungunya virus and West Nile Virus (WNV)^56^. Other RNA viruses trigger NLRP inflammasome assembly (influenza, dengue virus and hepatitis C)^57^, and RIG-I inflammasome (vesicular stomatitis virus and encephalomyocarditis virus)^57^. Once formed these inflammasomes lead to caspase-1 activation, a process promoted by Caspase Recruitment Domain Family Member 16 (CARD16)^58, 59^. Pro-caspase-1 is cleaved to its activated form, caspase-1 p20 subunits, which in turn activates inflammatory cytokines, Interleukin (IL)-18 and IL-1β in addition to inducing pyroptosis^46, 60^.

To date, there is no evidence of AIM2 inflammasome formation and pyroptosis in EV-A71 infection. In this study, we first performed microarray/trancriptome analysis on human neuroblastoma (SK-N-SH) cells following EV-A71 infection. Based on findings that suggested involvement of AIM2 inflammasome, we hypothesized that pyroptosis may be an important mechanism in EV-A71-induced neuronal cell death. We further performed RT-qPCR analysis, western blotting, immunofluorescence and flow cytometry in infected cells, and immunohistochemistry on human autopsy tissues of EV-A71 encephalomyelitis to confirm this. We found that AIM2 and caspase-1 upregulation reduces EV-A71 replication by inducing pyroptosis in SK-N-SH cells. AIM2 expression in human CNS tissues of EV-A71 encephalomyelitis was also demonstrated. Our results highlight the importance of AIM2 inflammasome and pyroptosis in EV-A71 neuronal infection.

## Results

### Transcriptome analysis of EV-A71 infection in SK-N-SH cells

**Figure 1:**
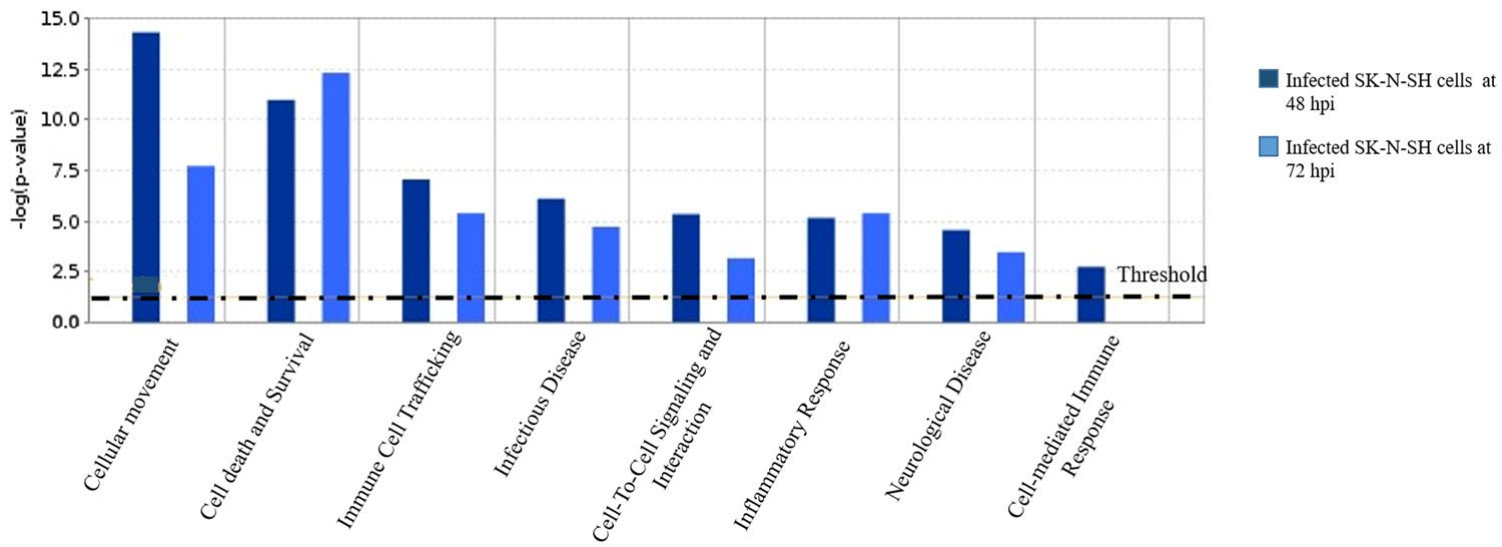
Disease and function pathways derived from transcriptome and Ingenuity pathway analysis of infected SK-N-SH cells. The most highly up-regulated disease and function genes involved cellular movement and cell death/survival. The graph shows the category scores where the “threshold” indicates the minimum significance level [calculated as -log (*p*-value) using the Fisher’s exact test, set at 1.25].

**Figure 2:**
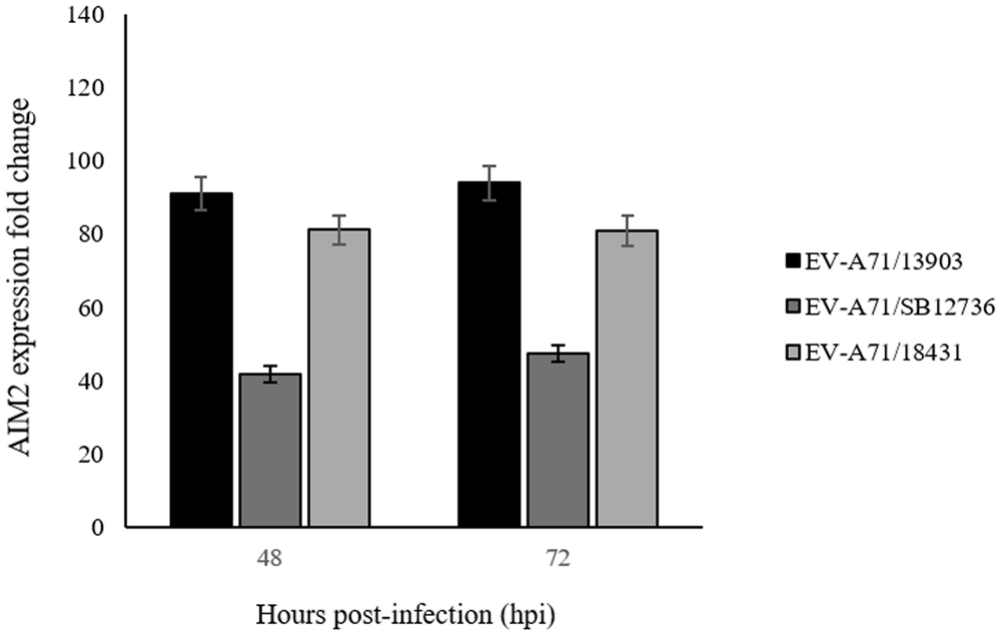
EV-A71 strains up-regulated AIM2 expression in infected SK-N-SH cells. AIM2 expression in infected SK-N-SH cells determined by real-time PCR analysis in all three EV-A71 strains (13903, 18431, and SB12736) showed significantly higher AIM2 fold change relative to mock-infected controls at 48 and 72 hpi. All data represent the mean ± standard deviation of a set of triplicates.

We analyzed the effect of EV-A71/13903 infection in human SK-N-SH cells at the gene expression level when >50% infection was achieved (Supplementary Figure S1). Among 24,000 genes analyzed, pairwise comparisons between infected and uninfected cells at ≥2-fold change threshold, showed 287 up-regulated genes and 390 down-regulated genes at 48 hpi, and 702 up-regulated and 674 down-regulated genes at 72 hpi (Gene expression omnibus accession: GSE71673). These genes were classified according to their molecular and cellular functions using the Ingenuity pathway analysis software, which showed that the most dysregulated genes involved cellular movement, and cell death and survival (Fig. 1). Among the most highly up-regulated genes, only AIM2 is associated with cell death/survival, while other genes were associated with cellular movement, cell death/survival or other functions (Table 1). Up-regulation of downstream of AIM2-mediated, pyroptosis-associated genes, CARD16 and caspase-1 was also observed (Table 1). However, IL-1β showed a 1.98 fold change while IL-18 expression was not detected. To confirm that AIM2 up-regulation was not restricted to EV-A71/13903, RT-qPCR was performed on EV-A71/18431 and EV-A71/SB12736 infected SK-N-SH cells, and the results show AIM2 up-regulation as well (Fig. 2).

**Table 1:** Selected up-regulated genes in EV-A71/13903 infected SK-N-SH cells at 48 and 72 hours post-infection (hpi).

48 hpi			72 hpi		
Fold Change	ANOVA p-value	Gene	Fold Change	ANOVA p-value	Gene
**Topmost-upregulated genes ranked by degree of fold change**					
13.35	0.044962	Chemokine (C-X-C motif) ligand 11 (CXCL11)	15.26	0.003415	Serine peptidase inhibitor, Kazal type 6
9.3	0.039725	Cathepsin S	14.13	0.016601	Chemokine (C-C motif) ligand 5
9.17	0.000695	superoxide dismutase 2, mitochondrial (CSF2)	13.73	0.000022	Deleted in lymphocytic leukemia 2 (non-protein coding)
**7.92**	**0.000113**	**Absent in melanoma 2 (AIM2)**	9.96	0.000025	Colony stimulating factor 2 (granulocyte-macrophage)
7.4	0.005032	Tumor necrosis factor, alpha-induced protein 6 (TNFAIP6)	8.99	0.015315	Chemokine (C-X-C motif) ligand 11
7.31	0.036801	Interleukin 8 (IL-8)	7.2	0.000796	Tumor necrosis factor, alpha-induced protein 6
6.95	0.026789	Zinc finger CCCH-type, antiviral 1	7.17	0.000134	Family with sequence similarity 111, member B (FAM111B)
6.56	0.01899	Chemokine (C-C motif) ligand 20 (CCL20)	6.86	0.000719	Cathepsin S
6.47	0.040204	Chemokine (C-C motif) ligand 5 (CCL5)	6.67	0.000206	superoxide dismutase 2, mitochondrial
5.57	0.00589	Laminin, beta 3	6.64	0.006316	Chemokine (C-C motif) ligand 5
5.39	0.030611	Colony stimulating factor 2 (granulocyte-macrophage) (CSF2)	6.29	0.000198	Family with sequence similarity 111, member B
5.08	0.002999	Interleukin 6 (interferon, beta 2) (IL-6)	6.25	0.000991	Interleukin 8
5	0.01762	Interferon-induced protein with tetratricopeptide repeats 2	**6.19**	**0.00103**	**Absent in melanoma 2 (AIM2)**
4.91	0.039786	SP100 nuclear antigen	5.96	0.009354	Chemokine (C-C motif) ligand 20
4.89	0.015657	Tumor necrosis factor, alpha-induced protein 3 (TNFAPI3)	5.7	0.000114	Chemokine (C-X-C motif) ligand 3
4.84	0.025299	Cathepsin S	5.59	0.001701	Cell division cycle 25 A
4.37	0.012194	Serine peptidase inhibitor, Kazal type 6	5.58	0.01762	Chemokine (C-X-C motif) ligand 10 (CXCL10)
4.31	0.042266	Chemokine (C-X-C motif) ligand 3 (CXCL3)	5.28	0.003336	Chemokine (C-X-C motif) ligand 1
**AIM2-mediated, pyroptosis-associated genes**					
3.02	0.030757	Caspase 1 (CASP1)	2.16	0.009802	Caspase recruitment domain family, member 16 (CARD16)
2.12	0.031591	Caspase recruitment domain family, member 16 (CARD16)	2.01	0.016223	Caspase 1 (CASP1)
ND	ND	Interleukin 1, beta (IL-1β)	1.98	0.672011	Interleukin 1, beta (IL-1β)

The fold change threshold used to determine gene up-regulation was ≥2.00 relative to mock-infected cells. ANOVA p-values were derived from the two-way ANOVA statistical test: p ≤ 0.05 is significant. AIM2 fold change was 7.92 at 48 hpi and 6.19 at 72 hpi (**highlighted in bold**).

*ND = not detectable.

### RT-qPCR validation of AIM2, CARD16, Caspase-1 and IL-1β up-regulation in infected SK-N-SH cells

**Figure 3:**
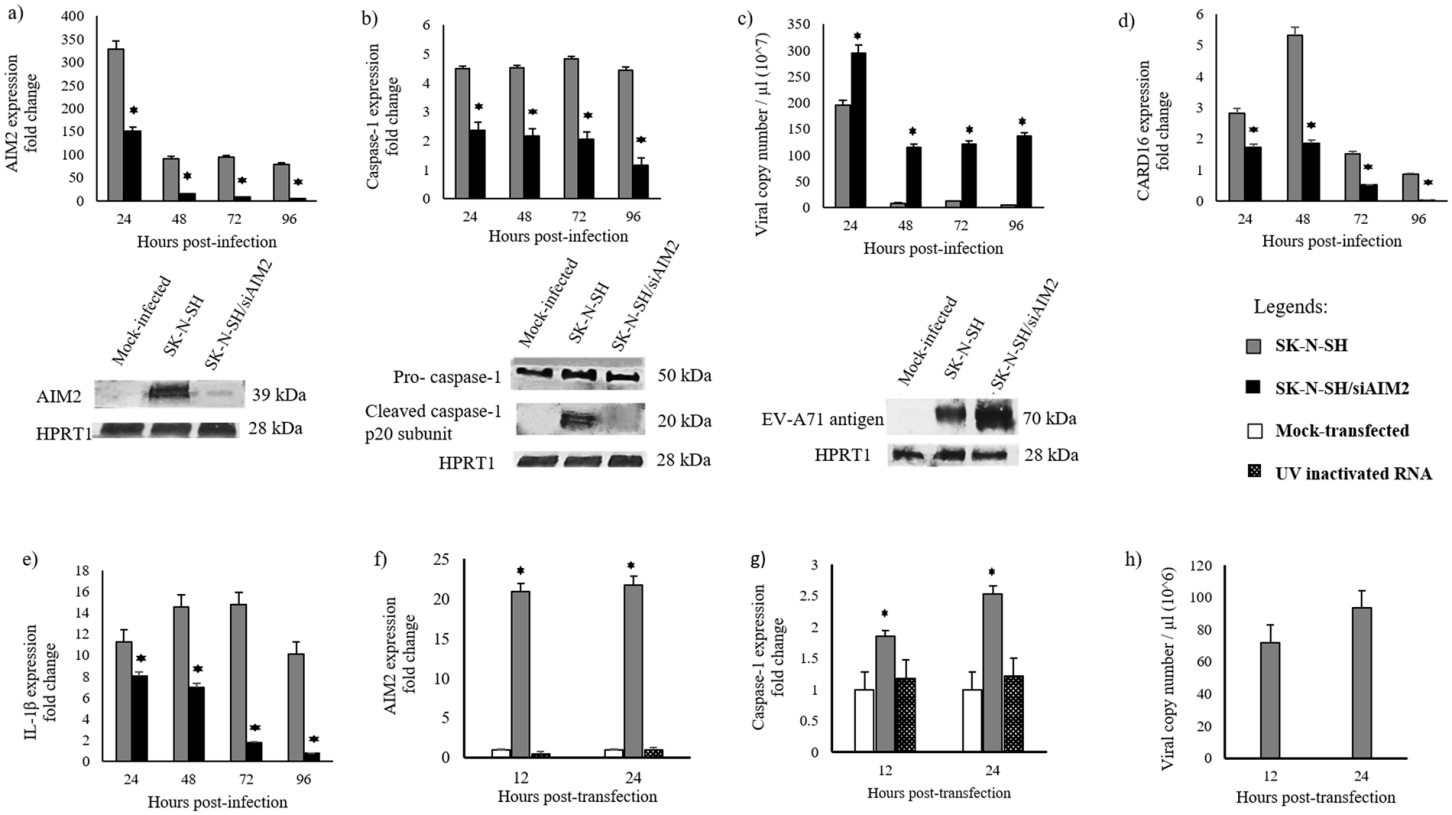
AIM2, CARD16, caspase-1 and IL-1β up-regulation, caspase-1 activation and viral replication inhibition was observed in infected-SK-N-SH cells but not in SK-N-SH/siAIM2 cells. There was up-regulation of AIM2 (**a**), caspase-1 (**b**), CARD16 (**d**) and IL-1β (**e**) expressions in infected SK-N-SH cells, and significant reduction (*p < 0.05) in SK-N-SH/siAIM2 cells. Western blot analysis showed a marked reduction/absence of AIM2 protein (**a**) and cleaved caspase-1 p20 subunits (activated caspase-1). Cropped blots are presented due to different primary and secondary antibody procedures during staining. (**b**) Viral copy numbers (**c**) were about 10 fold higher (*p < 0.05) in infected SK-N-SH/siAIM2 cells for all time points, corresponding to Western blot analysis that showed much higher viral proteins in infected cells (**c**). AIM2 (**f**) and caspase-1 (**g**) gene expressions in EV-A71/13903 viral RNA transfected SK-N-SH cells showed significant up-regulation (*p < 0.05) at 12 and 24 hpt, respectively. UV-inactivated EV-A71/13903 RNA transfection showed no up-regulation of AIM2 (**f**) and caspase-1 (**g**) gene expression at both time points. Viral copies at 12 to 24 hpt in viral RNA transfected SK-N-SH cells were detected but the apparent differences were not statistically significant (p = 0.7009) (**h**). All data represent the mean ± standard deviation of a set of triplicates.

Reconfirmation of AIM2 expression and up-regulation of downstream pyroptosis-associated genes CARD16, caspase-1 and IL-1β was validated using RT-qPCR (Fig. 3). At 48 and 72 hpi, consistent with, but much higher than transcriptome results, AIM2 was up-regulated by 90 and 105 folds, respectively, compared to mock-infected cells (Fig. 3a). At the additional time points of 24 and 96 hpi, the AIM2 fold change was 320 and 80, respectively. Overall, AIM2 fold change was maximum at 24 hpi. From 48 to 96 hpi, AIM2 expression dropped slightly and plateaued.

Caspase-1 expression was uniformly increased by 4–5 folds for all time points (Fig. 3b). The CARD16 expression was increased at 24, 48 and 72 hpi but not at 96 hpi (Fig. 3d). Thus, RT-qPCR results confirmed our transcriptome analysis showing AIM2, CARD16 and caspase-1 up-regulation in EV-A71/13903-infected SK-N-SH cells. Furthermore, in contrast to transcriptome results, IL-1β showed a significant 10–15 folds increase for all time points (Fig. 3e).

To determine that EV-A71 viral RNA alone was also able to trigger AIM2 up-regulation, we transfected EV-A71/13903 RNA into SK-N-SH cells. AIM2 gene was highly expressed (21 fold) at 12 and 24 hpt (22 fold) (Fig. 3f), in tandem with elevation of caspase-1 expression at 12 (2 fold) and 24 hpt (3 fold) (Fig. 3g) compared to mock-transfected cells. EV-A71/13903 viral copies were detected from 12 to 24 hpt (Fig. 3h). Transfection of SK-N-SH cells using UV inactivated viral RNA did not up-regulate AIM2 expression at 12 hpt (0.9 folds) and 24 hpt (1.2 folds), and caspase-1 expression at 12 hpt (1.2 folds) and 24 hpt (1.1 folds) (Fig. 3f and g).

### AIM2 knockdown reduced caspase-1, CARD16, IL-1β and increased viral replication

Infected AIM2 knockdown (SK-N-SH/siAIM2) cells showed a marked decrease (>50%) of AIM2 expression from 24 to 96 hpi with the lowest levels after 24 hpi (Fig. 3a). Western blot analysis confirmed that AIM2 proteins were nearly undetectable at 72 hpi (Fig. 3a). There was significant concomitant decrease of CARD16 (p < 0.05, Fig. 3d) and caspase-1 (p < 0.05, Fig. 3b) expression for all time points. By Western blot analysis, cleaved caspase-1 p20 subunits (activated caspase-1) was absent in infected SK-N-SH/siAIM2 cells but pro-caspase-1 (unactivated caspase-1) was present (Fig. 3b). Moreover, significant decrease of IL-1β expression (Fig. 3e) for all time points was observed. The positive (siRNA GAPDH knockdown) and siRNA negative controls showed expected results (Supplementary Figure S2a). The results showed transfection efficiency of siRNA of 86% and 98% as shown in siGLO-expressing SK-N-SH cells at 72 and 96 hpt, respectively, was considered acceptable. 7-AAD positivity was low (ranging from 1 to 8%) at 24 to 144 hpt (Supplementary Figure S2b).

**Figure 4:**
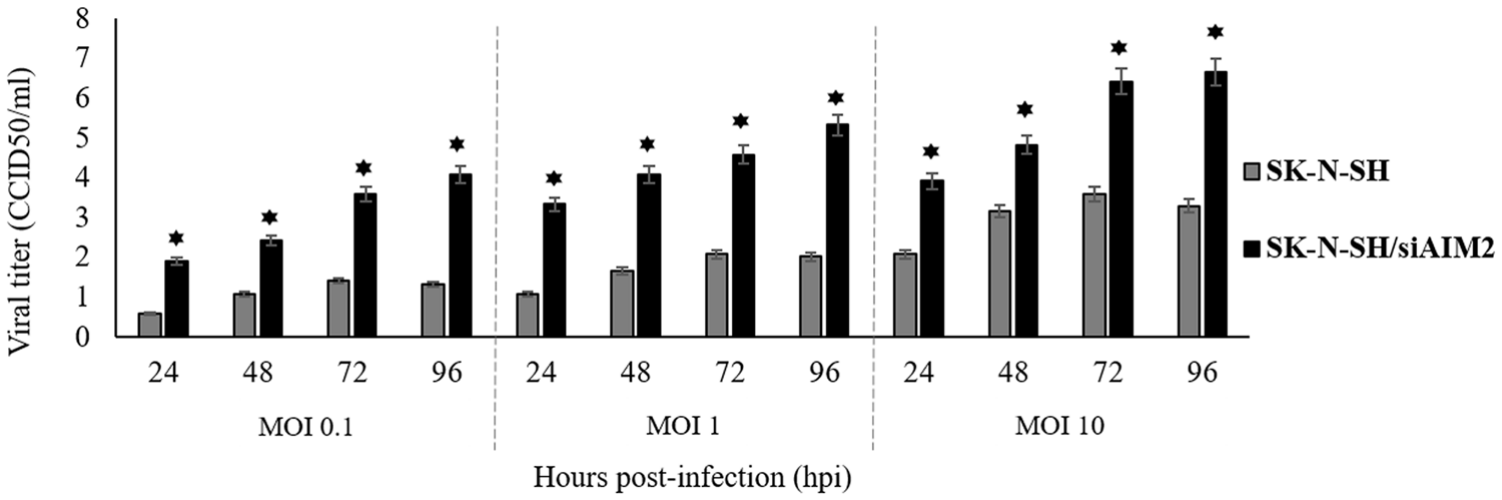
Viral replication inhibition did not depend on multiplicity of infection. (**a**) Viral titration of EV-A71/13903-infected SK-N-SH and SK-N-SH/siAIM2 cells at MOIs of 0.1, 1 and 10. Significantly lower (*p < 0.05) viral titers were observed in infected SK-N-SH cells compared to SK-N-SH/siAIM2 for all time points at all MOIs. In SK-N-SH/siAIM2 cells, a gradual increase in viral titers were observed from 24 to 72 hpi, while in SK-N-SH cells no significant increase was observed after 48 hpi. All data represent the mean ± standard deviation of a set of triplicates.

In contrast to SK-N-SH cells, infected SK-N-SH/siAIM2 cells showed significantly higher viral RNA copies (10 fold higher; p < 0.05) (Fig. 3c), viral titers (~2 folds overall; p < 0.05) for all time points (Fig. 4), and much higher viral antigens at 72 hpi (Fig. 3c) by Western blot analysis. At lower MOIs of 0.1 and 1, viral titers were also significantly higher (~2 folds overall; p < 0.05) in SK-N-SH/siAIM2 cells, suggesting that the pattern of viral replication is the same regardless of the size of inoculum (Fig. 4).

**Figure 5:**
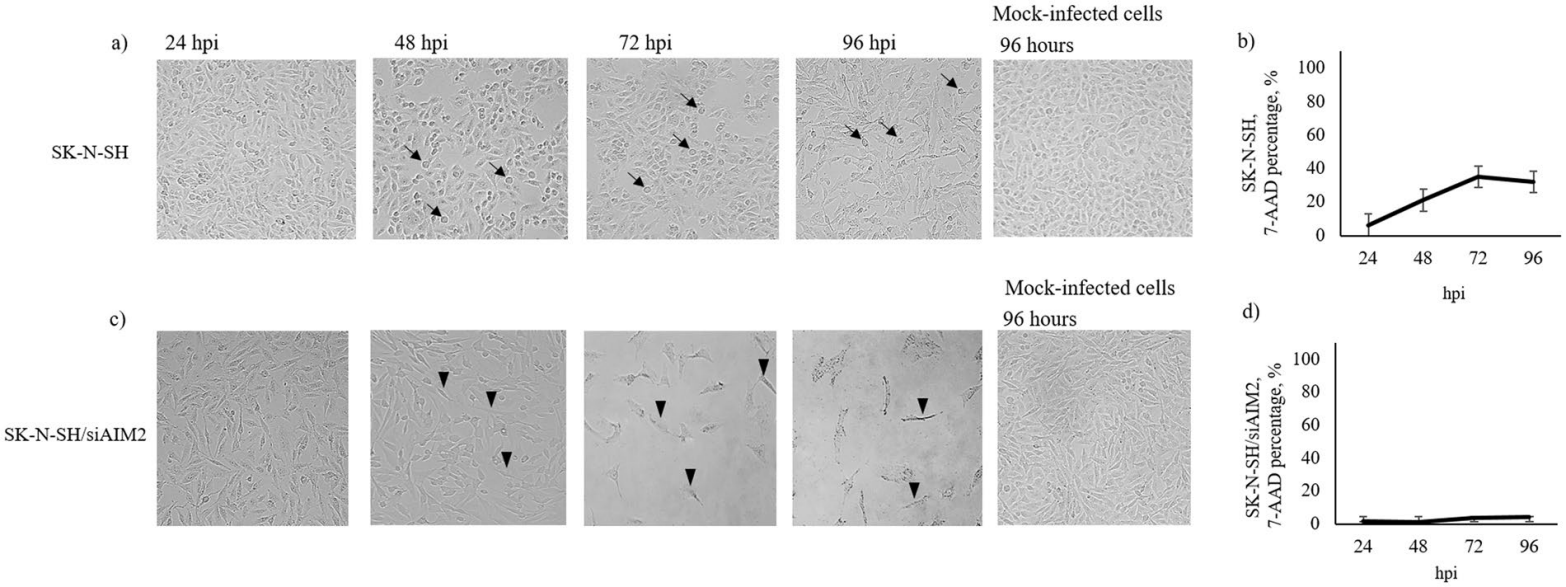
Cell swelling correlated with 7-AAD staining in infected SK-N-SH and SK-N-SH/siAIM2 cells. (**a**) Cellular morphology of EV-A71/13903-infected SK-N-SH cells at MOI of 10 showed pyroptotic cell swelling (arrows) from 48 hpi. (**b**) Flow cytometry analysis of percentage of the 7-AAD positive infected SK-N-SH cells showed significant increase from at 24 hpi onwards. (**c**) In contrast, SK-N-SH/siAIM2 cells showed extensive cell shrinkage (arrow heads) and detachment rather than swelling from 48 hpi onwards. (**d**) This correlated with the percentage of 7-AAD stained infected SK-N-SH/siAIM2 cells was low at about 6% throughout. All data represent the mean ± standard deviation of a set of triplicates.

Cell swelling suggestive of pyroptosis was observed only in infected SK-N-SH cells from 48 hpi onwards but not in infected SK-N-SH/siAIM2 cells (Fig. 5a). This result correlated with flow cytometry analysis of 7-AAD staining in infected SK-N-SH cells which showed significant increase at 48 hpi (Fig. 5b). Conversely, cell shrinkage and detachment were observed in infected SK-N-SH/siAIM2 cells from 48 hpi (Fig. 5c). Similarly, flow cytometry analysis of infected SK-N-SH/siAIM2 cells showed only about 6% of 7-AAD positivity throughout (Fig. 5d).

Taken together, the data suggest that AIM2 knockdown reduced CARD16, activated caspase-1 and IL-1β, resulting in amelioration of AIM2 inflammasome suppression of EV-A71 replication in SK-N-SH cells.

### Co-localization of viral and AIM2 antigens in infected SK-N-SH and SK-N-SH/siAIM2 cells

**Figure 6:**
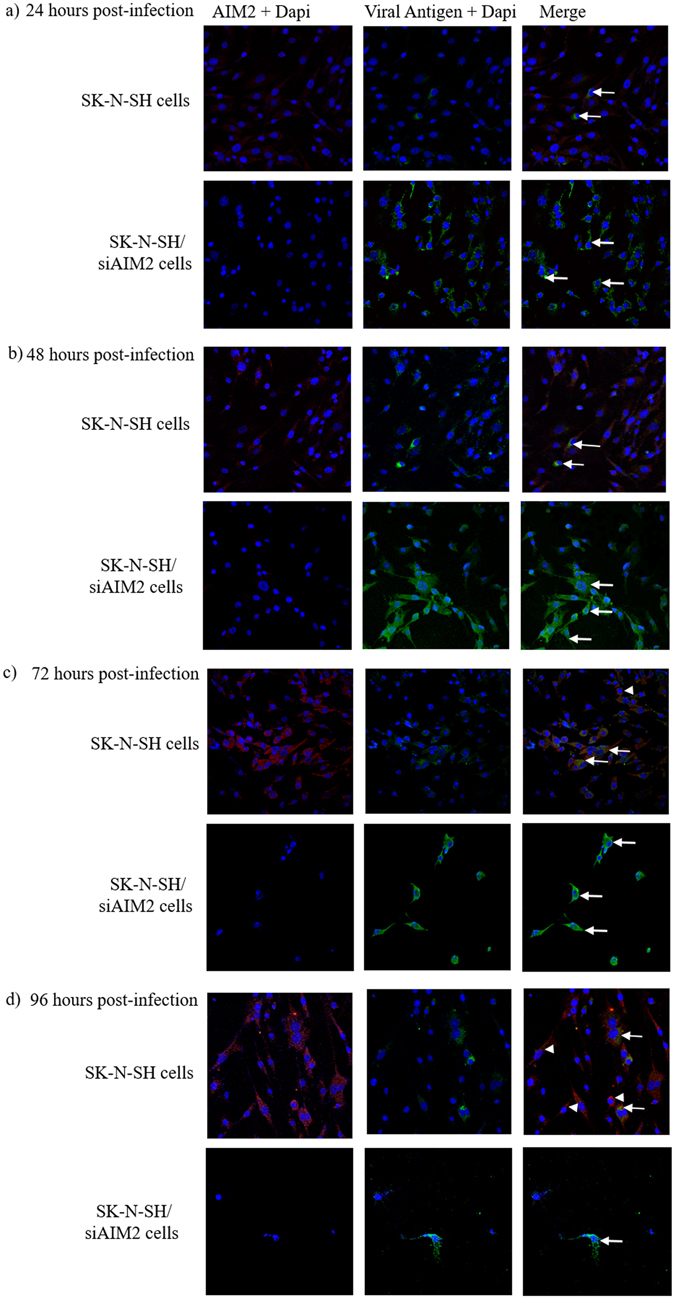
Viral infection induced AIM2 and pyroptosis in SK-N-SH cells but not in SK-N-SH/siAIM2 cells. EV-A71/13903-infected SK-N-SH cells and SK-N-SH/siAIM2 cells at 24 (**a**), 48 (**b**), 72 (**c**) and 96 hpi (**d**), stained for viral antigens (green), AIM2 (red) and dapi (blue, nuclear staining). Viral antigen-positive SK-N-SH cells were also positive for AIM2 (**a**–**d**, white arrows) for all time points. At 72 and 96 hpi but not at earlier time points, viral antigen-negative cells stained positive for AIM2 (**c**,**d**, white arrowheads). SK-N-SH/siAIM2 cells showed extensive viral antigen staining ((**a**–**d**), white arrows) without AIM2 expression for all time points. Magnification 20x.

Generally, we were able to demonstrate co-localization of viral antigens and AIM2 protein in most infected cells by double IF at all time points (Fig. 6a–d; arrows) but mock-infected cells showed very little to no AIM2 positivity (data not shown). AIM2 expression was found to be maximum at 72 and 96 hpi in infected SK-N-SH cells. At these late time points, viral antigens were reduced or absent (arrowhead; Fig. 6c,d) compared to 24 and 48 hpi when viral antigens were more evident. In infected SK-N-SH/siAIM2 cells, AIM2 was absent in all cells at all time points. However, viral antigens gradually increased from 24 hpi, and almost all cells were viral antigen-positive by 72 and 96 hpi (Fig. 6a–d; arrows).

**Figure 7:**
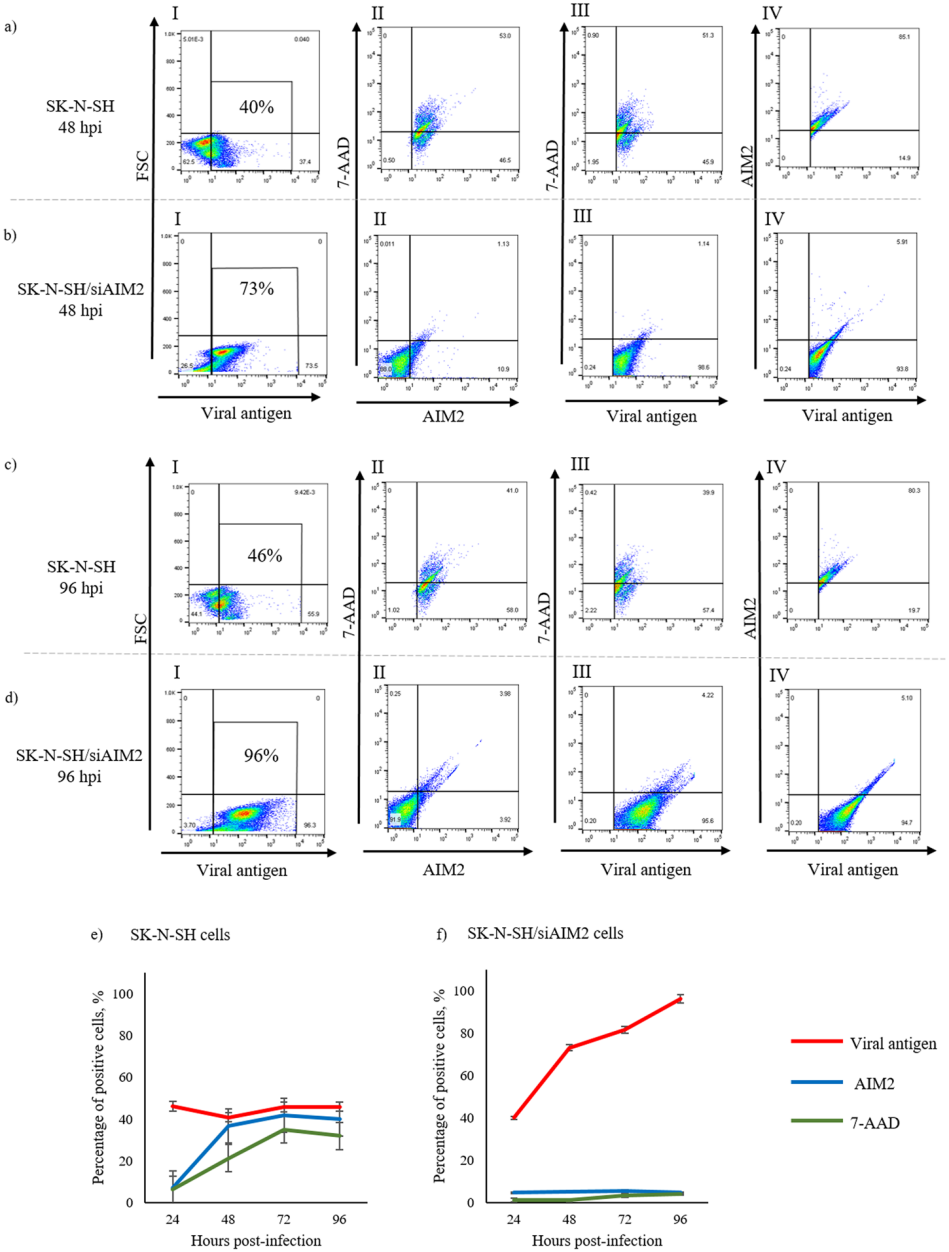
AIM2 induced pyroptosis limited viral replication in infected SK-N-SH but not in SK-N-SH/siAIM2 cells. Flow cytometry analysis of viral antigens, AIM2 expression and 7-AAD staining in infected SK-N-SH and SK-N-SH/siAIM2 cells as acquisition plots, at 48 (**a**,**b**) and 96 (**c**,**d**) hours post-infection (hpi), and as percentage graphs at 24, 48, 72 and 96 hpi (**e**,**f**). (**a**,**b**) At 48 hpi, viral antigen-positive SK-N-SH cells at about 40% (**a**,I) was significantly lower (p = 0.0002) compared to SK-N-SH/siAIM2 cells at about 73% (**b**,I). SK-N-SH cells with viral antigen, AIM2 and 7-AAD positivity at about 53% (**a**,II) was significantly much higher (p = 0.0002) than in SK-N-SH/siAIM2 cells, which was about 1% (**b**,II). Among viral antigen-positive SK-N-SH cells, 7-AAD positivity of about 51% (**a**,III) was significantly much higher (p = 0.0001) than in SK-N-SH/siAIM2 cells which was about 1% (**b**,III). Similarly, among viral antigen-positive SK-N-SH cells, AIM2 positivity was significantly much higher (p = 0.003) at about 85% (**a**,IV) than in SK-N-SH/siAIM2 cells, which was about 6% (**b**,IV). (**c**,**d**) A similar pattern was observed in SK-N-SH cells at 96 hpi, in which viral antigen-positivity (about 46%) (**c**,I) was significantly higher (p = 0.0009) than in SK-N-SH/siAIM2 cells (about 96%) (**d**,I). SK-N-SH cells with viral antigen, AIM2 and 7-AAD positivity (about 41%) (**c**,II) were significantly much higher (p = 0.001) than SK-N-SH/siAIM2 cells (about 4%) (**d**,II). The same pattern of significantly much higher (p = 0.001) viral antigen/7-AAD positive SK-N-SH cells (**c**,III), and significantly much higher (p = 0.006) viral antigen/AIM2 positive cells (**c**,IV) compared to SK-N-SH/siAIM2 cells (**d**,III and **d**,IV) was also observed. (**e**) The percentage of AIM2 and 7-AAD-positivite in SK-N-SH cells, showed significant increase (p = 0.003 and p = 0.0005, respectively) from 24 to 48 hpi but the percentage of viral antigen-positive cells did not increase at these and later time points. (**f**) In contrast, AIM2 and 7-AAD-positive SK-N-SH/siAIM2 cells were below 6% for all time points, while a sharp and significant increase of viral antigen-positive cells was observed from 24 hpi onwards (p = 0.007, 0.03 and 0.04 at 48, 72 and 96 hpi, respectively). All data represent the mean ± standard deviation of a set of triplicates.

Flow cytometry analysis further confirmed that infected SK-N-SH cells and demonstrated significantly lower percentage of viral antigens of about 40% (Fig. 7a,I) at 48 hpi (p = 0.0002), and about 46% (Fig. 7c,I) at 96 hpi (p = 0.0009) compared to infected SK-N-SH/siAIM2 cells and at about 73% (Fig. 7b,I) and 96% (Fig. 7d,I), respectively. In SK-N-SH cells, although there were fewer viral antigen-positive cells, about 85% and 80% at 48 (Fig. 7a,IV) and 96 hpi (Fig. 7c,IV), respectively, were AIM2 positive. However, in SK-N-SH/siAIM2 cells, despite the higher number of viral antigen-positive cells, AIM2 was positive in only about 6% and 5% of cells at 48 (Fig. 7b,IV) and 96 hpi (Fig. 7d,IV), respectively.

### AIM2 correlates with 7-AAD staining in infected SK-N-SH and SK-N-SH/siAIM2 cells

To determine loss of cell membrane integrity, an inherent feature of pyroptosis, we stained for 7-AAD in infected SK-N-SH cells. Cells triple-stained with 7-AAD, AIM2 and viral antigens were analyzed by flow cytometry to study their correlation. Results showed 7-AAD positivity in about 53% (48 hpi; Fig. 7a,II) and 41% (96 hpi; Fig. 7c,II) in AIM2-positive cells and about 51% (48 hpi; Fig. 7a,III) and 40% (96 hpi; Fig. 7c,III) of viral antigen-positive cells. In contrast, infected SK-N-SH/siAIM2 cells showed only about 1% (48 hpi; Fig. 7b,II) (p = 0.0002) and 4% (96 hpi; Fig. 7d,II) (p = 0.001) 7-AAD positivity in AIM2-positive cells, and about 1% (48 hpi; Fig. 7b,III) (p = 0.0001) and 4% (96 hpi; Fig. 7d,III) (p = 0.001) of viral antigen-positive cells. AIM2 protein was absent in mock-infected SK-N-SH cells (Supplementary Figure S3) confirming results shown by Western blot (Fig. 3a). Positive staining controls showed expected results (Supplementary Figure S3).

Figure 7e and f show graphs that correlate the percentage of infected cells that were AIM2, 7-AAD and viral antigen positive over various time points. In SK-N-SH cells (Fig. 7e), at 24 hpi, there was a low percentage of AIM2 and 7-AAD positivity (about 5%) while there was a high percentage of viral antigen positive cells (about 45%). However, at 48 hpi, a significant increase in AIM2 and 7-AAD positivity with a decrease in viral antigen positive cells were observed. At 72 and 96 hpi, percentage of AIM2, 7-AAD and viral antigen positive cells plateaued at about 40%. On the other hand, in SK-N-SH/siAIM2 cells (Fig. 7f), very low AIM2 and 7-AAD-positive cells were observed for all time points but there was significant increase in viral antigen-positive cells from 24 to 96 hpi. This suggests that pyroptosis (7-AAD positivity) occurs only in AIM2-expressing infected SK-N-SH cells, and was significantly reduced in SK-N-SH/siAIM2 cells, and that AIM2 induced pyroptosis inhibits viral replication in SK-N-SH cells.

### AIM2 was highly expressed in the CNS tissues of human EV-A71 encephalomyelitis patients

**Figure 8:**
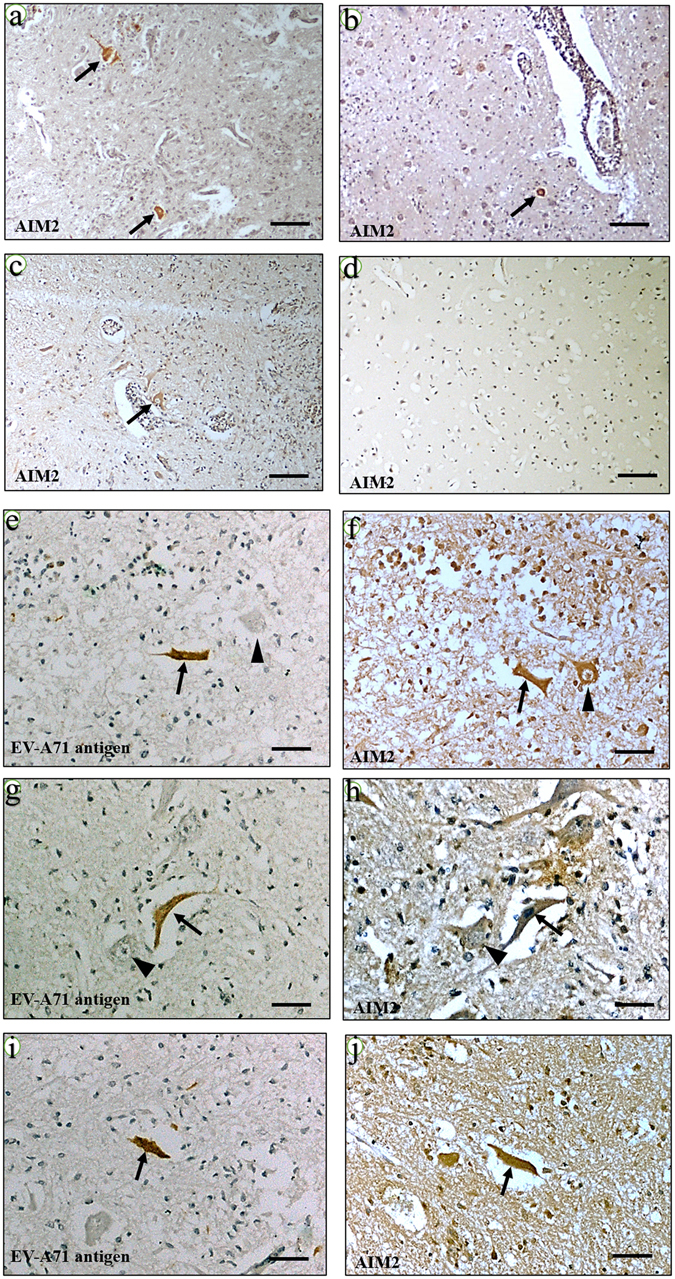
AIM2 antigens was expressed in inflamed areas and EV-A71-infected neurons in human encephalomyelitis. AIM2 was positive in inflamed areas of the spinal cord (**a**,**b**) and medulla (**c**) (arrows). In the respective, immediately adjacent spinal cord tissue sections, viral antigens ((**e**,**g**,**i**) arrows) and AIM2 ((**f**,**h**,**j**) arrows) were positive in the same neurons. Some neurons were AIM2 positive but viral antigen negative ((**e**,**f**,**g**,**h**) arrowheads). The cerebral cortex (**d**) and other uninflamed areas were negative for AIM2 and viral antigens. Immunohistochemistry using DAP and counter stained with hematoxylin. Magnification 20x (**a**–**d**) and 20x (**e**–**j**). Scale bar = 100 μm (**a**–**d**) and 50 μm (**e**–**j**).

To confirm the *in vitro* findings, IHC staining was performed to localize AIM2 protein in human CNS tissues of 3 autopsies. The spinal cord, medulla, pons, midbrain and the cerebral cortex were IHC stained with viral antigens or AIM2 protein (Fig. 8). AIM2-positive cells were detected in spinal cords (arrows, Fig. 8a,b) and medullas (arrows; Fig. 8c) only in the inflamed areas in all 3 cases. In one case, EV-A71 viral antigens (arrow; Fig. 8e,g and i) was demonstrated in the same neurons where AIM2 was positive (arrow, Fig. 8f,h and j), while some neurons were AIM2 positive but viral antigen negative (arrowheads, Fig. 8e,f and g,h). In all 3 cases there was no AIM2 staining in the cerebral cortex (Fig. 8d) and other regions where inflammation were absent.

## Discussion

Hitherto, the exact mechanism of neuronal cell death in EV-A71 encephalomyelitis remains unclear. Our study strongly suggests that AIM2 inflammasome-induced pyroptosis following EV-A71 infection is an important cell death mechanism in neurons. AIM2 plays a critical role in the downstream activation of caspase-1 and CARD16 leading to AIM2 inflammasome assembly that triggers pyroptosis and the activation of IL-1β and IL-18^61^. Activated IL-1β amplifies inflammatory response by stimulating immune cell activation^61^ while IL-18 stimulates production of IFN-γ^62^. Pyroptosis also releases other intra-cytoplasmic pro-inflammatory cytokines^63^.

We used a high throughput transcriptome platform and the Ingenuity pathway analysis to study gene expressions as initial screening of neuronal responses to infection, and found that the most up-regulated genes were involved in cell movement, cell death/survival and immune cell trafficking pathways (Fig. 1). We chosed to focus on AIM2 as it is the only highly up-regulated gene involved specifically in pyroptosis, a novel pro-inflammatory cell death mechanism characterized by caspase-1 activation, DNA breakages without laddering, cell swelling, plasma membrane rupture and pro-inflammatory cytokine release^41^. Recent studies have described the importance of inflammasomes in suppressing virus replication^57^, thus a better understanding may provide potential tools for intervention strategies in viral infections. Moreover, in the transcriptome analysis, other highly up-regulated genes were not related to cell death/survival processes such as apoptosis, autophagy or necroptosis.

AIM2 up-regulation was validated by RT-qPCR, which showed a marked increase of AIM2 gene expressions in all 3 EV-A71 strains (Figs 2 and 3a). AIM2 protein was detected in infected SK-N-SH cells of (Figs 3a and 6a–d and 7a and c), while totally absent in mock-infected cells at the 72 hpi (Figs 3a and S3). As part of the AIM2 inflammasome assembly and pyroptosis pathway, CARD16 and caspase-1 up-regulation (Table 1) were also confirmed by RT-qPCR for all time points (Fig. 3). Activated caspase-1 (cleaved caspase-1 p20 subunits)^46^, was detected only in infected cells (Fig. 3b). Interestingly, even though IL-1β was not shown to be highly up-regulated in the transcriptome analysis (Table 1), the RT-qPCR confirmed its up-regulation at all time points (Fig. 3e). However, IL-18 was not detected by transcriptome analysis. Infected SK-N-SH/siAIM2 showed about 50–85% AIM2 reduction (Fig. 3a) resulting in corresponding and significant decreases of CARD16 (Fig. 3d), caspase-1 (Fig. 3b) and IL-1β (Fig. 3e) expressions. In agreement with AIM2 up-regulation in infected SK-N-SH cells, AIM2 protein was detected in neurons and inflammatory cells in human EV-A71 encephalomyelitis (Fig. 8). AIM2-positive neurons were exclusively found in areas where EV-A71 antigens and/or intense inflammation were observed^20, 64^, suggesting that AIM2 was only highly expressed in infected and inflamed areas of the CNS.

Our results showed that AIM2-positive, infected SK-N-SH cells were highly positive for 7-AAD (Fig. 7a and c) and increased proportionately with AIM2 expression (Fig. 7e). Conversely, infected SK-N-SH/siAIM2 cells showed a significantly lower proportion of 7-AAD-positive cells (Fig. 7b,d and f). Loss of cell membrane integrity during pyroptosis enables 7-AAD to readily permeate into cells, thus this dye has been used as a marker to distinguish programmed cell death by pyroptosis from apoptosis^41, 65^. In addition, pyroptotic cell swelling widely observed in infected SK-N-SH cells (Fig. 5a) but not in infected SK-N-SH/siAIM2 cells (Fig. 5c), correlated well with 7-AAD staining of infected cells (Fig. 5b and d). Taken together, these results suggest that EV-A71 infection triggered AIM2 expression and AIM2-mediated up-regulation of specific genes leading to pyroptosis and neuronal cell death.

Our data strongly indicates that AIM2 inflammasome upregulation and pyroptosis suppresses EV-A71 infection and replication in neurons. Increased AIM2 expression at early time points (Figs 3a and 7e) was associated with a significant reduction in viral copy numbers after 24 hpi (Fig. 3c) and suppression of viral titer increase after 48 hpi (Fig. 4). Furthermore, there were reduced viral antigen-positive cells at 72 and 96 hpi (Fig. 6c and d). On the other hand, compared to SK-N-SH cells, infected SK-N-SH/siAIM2 cells generally showed significantly higher viral RNA copies (Fig. 3c), viral titers (Fig. 4), proportion of viral antigen-positive cells (Fig. 7f), and higher expressions of viral antigens by IF (Fig. 6a–d) and flow cytometry (Fig. 7b,d and f) for all time points. Interestingly, at 24 hpi even though AIM2 mRNA expression in SK-N-SH and SK-N-SH/siAIM2 cells (Fig. 3a) was the highest, viral copies remained highest suggesting AIM2 takes effect thereafter. Furthermore, we believe that knock-down of AIM2 expression takes effects only after 48 hpi and the partial AIM2 mRNA levels present at 24 hpi in SK-N-SH/siAIM2 cells (Fig. 3a) may still be sufficient to suppress viral copies which explains the significant reduction from 24 to 48 hpi (Fig. 3c). However, from 48 hpi onwards, when AIM2 reduction was lowest and constant in SK-N-SH/siAIM2 cells, viral copies actually increased (p < 0.05) (Fig. 3c). This is in agreement with viral titers in SK-N-SH/siAIM2 cells where a substantial increase was observed only after 48 hpi (Fig. 4). Nonetheless, apart from AIM2, other unknown factors/mechanisms may also contribute to viral suppression. Decreasing viral replication in SK-N-SH cells and increasing viral replication in SK-N-SH/siAIM2 cells at later time points were strongly associated with the significant increase and decrease, respectively, with 7-AAD positivity (Fig. 7e–f). This suggests that AIM2 inflammasome-mediated pyroptosis may play an important role in limiting EV-A71 replication in general as both mild HFMD and fatal encephalomyelitis viral isolates could infect neuronal cells and up-regulate AIM2 expression (Fig. 2). This idea is analogous to the recognition that infected non-inflammatory host cells undergo apoptosis leading to premature cell death and limitation of intracellular virus propagation and spread^66, 67^. It has been shown that *in vitro* and *in vivo* WNV-induced neuronal apoptosis limited CNS injury, viral replication and dissemination^68, 69^. Similarly, in vesicular stomatitis virus infection, apoptosis limited viral replication through involvement of perforin and granzymes^70^. Chikungunya virus and WNV infection in human dermal fibroblast cells have been reported to activate caspase-1 which limited viral replication, but the authors did not associate these findings with pyroptosis^56^. To our knowledge, pyroptosis as a cell death mechanism that could limit viral replication and dissemination has not been reported before. A previous study on *Burkholderia pseudomallei* showed that caspase-11 induced pyroptosis could inhibit intracellular bacterial growth^62^ even though there was no release of IL-1β. Our findings and hypothesis should be further investigated using primary human neuronal cultures and animal models including AIM2-knockout mouse models, which have been previously shown to increase murine cytomegalovirus virus replication^55^.

Pyroptosis releases activated IL-1β and other pro-inflammatory cytokines eventually leading to limitation of infection^40, 71^. In murine cytomegalovirus and vaccinia virus infections in macrophage cultures and mouse models, AIM2 inflammasome-associated pyroptosis has been described, and activated IL-1β was thought to play a critical role in host innate immunity against infection^72–74^. In our study, we showed significant increase in IL-1β expression by RT-qPCR (Fig. 3e), consistent with pyroptosis. Hence, apart from macrophages, neutrophils and NK cells, our results suggested that virus-infected neuronal cells may also up-regulate IL-1β to recruit immune cells, including NK cells, to the site of infection, and to trigger IFN-γ production. Interestingly, in an EV-A71 infection mouse model, viral proteases were able to overcome the effects of NLRP3 inflammasome activated IL-1β leading to increased viral replication^75^. Hence, the role of AIM2 inflammasome activated IL-1β in EV-A71 infections needs to be further investigated. We also showed increase of tumour necrosis factor, IL-6 and CXCL10 in the transcriptomic analysis (Table 1 and Supplementary Figure S4), which were also found in the cerebrospinal fluids of EV-A71 encephalomyelitis patients^76–79^. It is possible that these inflammatory mediators may be released from infected CNS neurons as part of pyroptosis.

Our study showed that AIM2 inflammasome assembly could be triggered by cytosolic EV-A71 RNA while previously it was mostly described in cytosolic DNAs^80^. We also showed AIM2 gene up-regulation and activation following viral RNA transfection of SK-N-SH cells (Fig. 3f). However, UV-inactivated viral RNA failed to trigger AIM2 (Fig. 3f) and caspase-1 (Fig. 3g) up-regulation possibly because of photochemical damage to genomic RNA^81–83^, suggesting that UV damaged RNA may not activate AIM2 inflammasome. However, the detailed mechanisms by which AIM2 specifically detects and interacts with EV-A71 RNA is unknown. In the presence of cytosolic DNAs, AIM2 is known to bind DNA directly through its ASC via its pyrin domain^84^ and the two adjacent oligonucleotide/oligosaccharide-binding folds in the C-terminal HIN-200 domain^63^. Even though, AIM2 up-regulation has been described in Chikungunya virus and WNV infections in human fibroblast cells, the ability of viral RNAs to trigger AIM2 up-regulation was not investigated^56^. In conclusion, we found that EV-A71 infection of neuronal cells induced up-regulation of AIM2 and inflammasome assembly leading to pyroptosis cell death and suppression of viral replication.

## Materials and Methods

### Virus and cells

All EV-A71 strains used in this study were clinical isolates (Table 2). The viruses were grown and titrated in Vero cells by the cell culture infectious dose 50 (CCID_50_) assay as described previously^85, 86^. Vero (ATCC-CCL-81) and SK-N-SH (ATCC-HTB-11) cell lines were propagated in Dulbecco’s modified Eagle’s medium, supplemented with 5% and 10% fetal bovine serum, respectively. Rhabdomyosarcoma cells (ATCC-CCL-136) were propagated in Roswell Park Memorial Institute medium, supplemented with 10% fetal bovine serum, and used as positive controls in the double immunofluorescence assay. All assays using SK-N-SH cells were done in triplicates, and all procedures using commercial kits/reagents followed manufacturers’ protocols unless stated otherwise.

**Table 2:** Virus strains used in experiments.

Virus designation	Passage number	Year of isolation	Disease/Isolation site	Gene accession number
EV-A71/13903	6	1997	Fatal HFMD/medulla oblongata	AY207648
EV-A71/18431	3	2006	Fatal HFMD/throat	NA
EV-A71/SB12736	3	2003	HFMD/NA	AY794025

*NA – not available; HFMD – Hand, foot and mouth disease

### EV-A71 infection of SK-N-SH cells

SK-N-SH cells in 2 ml microcentrifuge tubes were infected with EV-A71/13903 at a multiplicity of infection (MOI) of 10 for all experiments unless otherwise stated. After keeping in a shaking incubator at 37 °C for 2 hours followed by centrifugation at 400 × g, the cell pellet was washed twice with phosphate-buffered saline (PBS) before re-suspension in 500 μl of culture medium and seeding into 24-well plates. After incubation at 37 °C, infected cells and supernatant were harvested at 24, 48, 72 and 96 hours post-infection (hpi) for flow cytometry studies, and RNA extraction for microarray and quantitative real-time PCR analysis. Mock-infected cells/RNA as negative controls were prepared following the same procedure.

### Flow cytometry

#### Quantitation of viral antigens in infected SK-N-SH cells

Flow cytometry was used to quantitate the proportion of infected SK-H-SH cells by viral antigen detection. Briefly, infected and mock-infected cells were trypsinized and centrifuged at 400 × g in 1.5 ml microcentrifuge tubes. Cell pellets were fixed with Fluorofix buffer (Biolegend, USA) for 30 min at room temperature (RT) in the dark^87–89^, and PBS-washed twice before permeabilization with 0.005% Triton-X100 for 10 min. Cells were then incubated for 1 hr at RT with mouse monoclonal Enterovirus blend 3321 antibody (dilution 1:50; Merck, Germany), followed by goat-anti mouse IgG conjugated with Alexa-fluor 488 (Molecular Probes, USA) for 30 min at RT in the dark^90^. Cells were re-suspended in cell staining buffer (Biolegend, USA) before analysis using the FACS Canto II Flow cytometer (Becton Dickson, USA) and the Flowjo, Single Cell Analysis Software version 10 (FlowJo,USA).

#### Co-localization of viral antigens with AIM2 or 7-amino-actinomycin in infected SK-N-SH cells

EV-A71/13903-infected SK-N-SH and AIM2-knockdown SK-N-SH cells (SK-N-SH/siAIM2) cells at MOI of 1, and mock-infected cells were triple-stained for EV-A71 antigens, AIM2 and 7 amino-actinomycin (7-AAD). Previously shown to be useful as a biomarker for pyroptosis, 7-AAD is able to enter nuclear membranes to stain DNA following the loss of membrane integrity^41, 65, 91^. Briefly, infected and mock-infected cells were collected in 1.5 ml microcentrifuge tubes and incubated for 5 minutes with 7-AAD^87–89^. The cell pellets were then fixed with Fluorofix buffer and stained for viral antigen and AIM2 protein were using mouse monoclonal Enterovirus blend 3321 antibody and rabbit polyclonal AIM2 K-12 antibody (dilution 1:500; Santa Cruz, USA), followed by incubation with by goat-anti mouse IgG conjugated with Alexa-fluor 488 and goat anti-rabbit IgG conjugated with Alexa-fluor 546 (Molecular Probes, USA) for 30 min at RT in the dark^92^. Flow cytometry analysis to assess localization of viral antigens and AIM2 or 7-AAD was performed as before.

#### Assessment of transfection efficiency and 7-AAD staining in viral RNA transfected SK-N-SH cells

Transfection efficiency was assessed by flow cytometry after transfection of 75 pmol siGLO Green Transfection Indicator (D-001630-01-20; Dharmacon, USA) in treated and untreated SK-N-SH cells at 48 h post-transfection (hpt). To assess 7-AAD staining, SK-N-SH cells were incubated with 7-AAD for 5 minutes at RT before fixation in Fluorofix buffer and flow cytometry analysis.

### Transcriptome analysis of infected SK-N-SH cells

Total RNA was extracted from infected and mock-infected SK-N-SH cells at the 48 and 72 hpi using the RNeasy Plus Mini kit (Qiagen, Germany). Biotinylated cRNA were prepared and fragmented from 100 ng total RNA (GeneChip 3′ IVT Express Array, 2008–2010, Affymetrix), followed by hybridization of 12 ug of cRNA for 16 hr at 45 °C on the GeneChip. After washing and staining using the Affymetrix® GeneChip® Fluidics Station 450, the GeneChip was scanned using the GeneChip® Scanner 3000. Data were analyzed with the Expression console software (default analysis settings and the RMA processing algorithm). Statistical tests for gene level differential expression were performed using the Transcriptome Analysis Console (TAC) v2.0 software. All data sets were deposited into the Gene Expression Omnibus (Gene accession: GSE71673) using MIAME guidelines. Further analysis of gene expression and pathways was done by the IPA software (Ingenuity® Systems, http://www.ingenuity.com/).

### Real time quantitative PCR (RT-qPCR) of viral RNA and selected genes in infected SK-N-SH cells

To quantify viral RNA copies, Taqman primers (EV-A71-F: GCACAACTCACCATTGGA, EV-A71–R: GCTGTAGCATCGTCATCA) and probe (FAM-CACCATTACTACACAGGAGGCGGC-TAMRA) were used. Total RNA was extracted from EV-A71/13903, EV-A71/18431 and EV-A71/SB12736 infected and mock-infected SK-N-SH cells at the 24, 48, 72 and 96 hpi using the RNeasy Plus kit (Qiagen, Netherlands). The cDNA template (1 µg), prepared using the iScript cDNA synthesis kit (Biorad, USA), was mixed with Taqman advance fast master mix (Applied Biosystem, USA), Taqman primers and probe. The cycling conditions were denaturation at 95 °C for 5 sec, amplification and quantification for 45 cycles at 95 °C for 30 s and 60 °C for 10 s, using the ABI 7500 fast real-time PCR system (Applied Biosystem, USA). The results were analyzed using the 7500 Software version 2.0.6. (Applied Biosystem, USA).

Based on transciptome analysis results, selected genes of interest, AIM2 (Hs00915710), caspase-1 (Hs00354836), CARD 16 (Hs03008439), and IL-1β (Hs00174097), were further investigated by RT-qPCR using commercially-available Taqman assays/reagents (Applied Biosystem, USA) and conditions, as described. Hypoxanthine phosphoribosyltransferase 1 (HPRT1; Hs02800695) and succinate dehydrogenase complex flavoprotein subunit A (SDHA; Hs00188166) were used as internal controls. Glyceraldehyde 3-phosphate dehydrogenase (GAPDH; Hs02786624) was used as siRNA control.

### AIM2 and caspase-1 gene expressions, and viral copy number quantitation in viral RNA transfected SK-N-SH cells

To study AIM2 and caspase-1 gene expressions following viral RNA transfection, SK-N-SH cells (5 × 10^4^ cells/ml) were seeded into 24-well plates at 37 °C overnight before transfection with 200ng of EV-A71/13903 viral RNA and UV-inactivated EV-A71/13903 viral RNA using Lipofectamine 2000 (Invitrogen, USA). Total RNA were extracted from transfected and mock-transfected cells at 12 and 24 hpt and RT-qPCR was performed as before. Viral RNA was inactivated by ultraviolet (UV) light as described previously^93^. Briefly, PBS-diluted virus stock was exposed to UV light for 30 minutes, and viral RNA extracted using High pure viral RNA extraction kit (Roche, Switzerland). Successful viral RNA inactivation was determined by absence of CPE and negative virus isolation in transfected Vero cells after 7 days. The transfection efficiency of Lipofectamine 2000 into cells was determined using 200 ng of Monster Green Fluorescent expression vector, mGFP (Promega, USA).

### AIM2 gene knockdown in SK-N-SH cells and EV-A71 infection

To further investigate its role in EV-A71 infection, AIM2 in SK-N-SH cells was silenced using Silencer® Select AIM2 siRNA (s18093; Ambion, USA) before infection. Briefly, SK-N-SH cells (5 × 10^4^ cells/ml) were seeded into 24-well plates at 37 °C overnight, before transfection with 75 pmol siRNA using RNAimax (Invitrogen, USA). At 48 hpt, SK-N-SH/siAIM2 cells were infected with EV-A71/13903. Cells were collected at 24, 48, 72 and 96 hpi for RT-qPCR to quantitate AIM2, CARD16, caspase-1 and IL-1β mRNA expression and viral RNA copies. Cells at 72 hpi were collected for Western blot analysis of these proteins. Silencer select GAPDH gene (Ambion, USA) and Silencer select Negative controls (Ambion, USA) were used as positive and negative internal controls, respectively. Transfection efficiency and cell viability post-transfection were analyzed using flow cytometry as before.

### Western blot analysis of AIM2 protein

After 72 hpi, infected and mock-infected SK-N-SH and SK-N-SH/siAIM2 cells were rapidly washed with ice-cold PBS, scraped and centrifuged at 1000 × g for 10 min. Total proteins were extracted using Protein EX cell lysis buffer (GeneAll, Korea) supplemented with a phosphatase inhibitor, Pierce phosphotase inhibitor mini table (Thermo, USA) to prevent protein degradation and preserve their activation states. Protein concentration was determined by the Pierce BCA reagents (Thermo, USA), and 20 μg protein supernatant fractions were denatured and subjected to SDS-PAGE as described previously^94^, with minor modifications. Briefly, the polyvinylidene fluoride membrane was incubated with AIM2 K-12 antibody (dilution 1:250; Santa Cruz, USA), Pierce EV-A71 Antibody (dilution 1:40000; Thermo, USA), caspase-1 antibody (dilution 1:100; Cell Signaling, USA), and HPRT1 antibody (dilution 1:500; Thermo, USA), respectively, overnight at 4 °C. Secondary goat anti-mouse IgG (31322) and goat anti-rabbit IgG (31342) antibodies conjugated with alkaline phosphatase (dilution 1:20000; Thermo, USA) were sequentially added. Blots were developed using 1-Step NBT/BCIP (Thermo, USA) at RT.

### Double immunofluorescence (IF) to detect AIM2 and viral antigens

Double IF was performed to co-localize of AIM2 and viral antigens in infected SK-N-SH and SK-N-SH/siAIM2. Approximately, 5 × 10^4^ cells were seeded onto Lab-Tek 8-well chamber slides (Nunc, Denmark) for 24 hours before infection with EV-A71/13903 for 2 hours at 37 °C. Infected and mock-infected cells were then fixed with methanol for 10 min at −20 °C and incubated with primary antibodies (AIM2-K12, dilution 1:500 and Enterovirus blend 3321, dilution 1:50) diluted in Tris-buffered-saline (TBS, pH 7.6) overnight at 4 °C. Secondary antibody staining as described before in the flow cytometry analysis, followed by DAPI counter staining was done before mounting in fluorescence mounting medium (Dako, Denmark). All the stained slides were analyzed using TCS SP5 II, Leica confocal laser scanning microscope (Leica, Germany). Normal human small intestine tissues were used as positive controls for AIM2. EV-A71/13903-infected Rhabdomyosarcoma cells served as positive controls.

### Immunohistochemistry (IHC) to detect viral antigens and AIM2 protein in human EV-A71 encephalomyelitis

The CNS tissues of 3 confirmed EV-A71 encephalomyelitis cases from a previous autopsy series^22, 95^ were investigated by IHC for AIM2 protein expression. IHC was performed by a standard ENVISION technique as described previously^96^. Briefly, deparaffinized and rehydrated tissue sections were endogenous peroxidase blocked before antigen retrieval at 30 min in Tris-EDTA buffer (pH 9) with 0.05% Tween-20. Tissue sections were then incubated with 10% normal goat serum before incubation overnight at 4 °C with mouse monoclonal Enterovirus blend 3321 antibody (dilution 1:50; Merck, Germany) or rabbit polyclonal AIM2 K-12 antibody (dilution 1:500; Santa cruz, USA). Secondary goat-anti mouse IgG HRP-conjugated (Dako, Denmark) or goat-anti rabbit IgG HRP conjugated (Dako, Denmark) respectively, was applied, followed by DAB (Dako, Denmark) chromogen. The slides were counterstained with hematoxylin (Dako, Denmark) and mounted with DPX mounting medium (Sigma, USA). Negative controls for IHC were normal human brain tissues. Positive controls for AIM2 staining were normal human small intestine and for EV-A71 antigens, EV-A71/13903 infected SK-N-SH cells. Duplicate assays on test tissues were also done by replacing the primary antibodies with isotype control antibodies or normal rabbit immunoglobulin fractions (l) (Dako, Denmark).

### Statistical analysis

Data was reported as mean ± standard deviation of at least three independent experiments performed. The unpaired student t-test in the Statistical Package for the Social Sciences (SPSS) was used to calculate p values. P values of ≤0.05 were considered significant.

### Ethics statement

All samples used in the experiments were from an already-existing collection and anonymized. The EV-A71/13903 virus ethics approval were obtained from Faculty of Medicine, Institutional Animal Care and Use Committee, University of Malaya (Ethics reference no: 2014-02-14/PATH/R/WKT). The viruses EV-A71/18431 and EV-A71/SB12736 were part of a HFMD surveillance program in collaboration of Sarawak Health Department with The Institute of Health and Community Medicine, University of Malaysia Sarawak where the Malaysia samples were given for research purposes. As such, an ethics or IRB approval was not needed.

## Electronic supplementary material

Supplementary Information
