# Supplementary material for: AIM2 Inflammasome-Mediated Pyroptosis in Enterovirus A71-Infected Neuronal Cells Restricts Viral Replication
Source: Sci Rep. 2017 Jul 19;7:5845. doi: 10.1038/s41598-017-05589-2 (PMC5517550; doi:10.1038/s41598-017-05589-2)
Supplement: Supplementary file 1 — Supplementary Information [file 41598_2017_5589_MOESM1_ESM.pdf]

## Title

AIM2 Inflammasome-Mediated Pyroptosis in Enterovirus A71-Infected Neuronal Cells Restricts Viral Replication


## Authors

Thinesshwary Yogarajah, BSc [1], Kien Chai Ong PhD [2], David Perera PhD[3] and Kum Thong Wong MBBS, Mpath, FRCPath [1]

## Author information

[1]From the Department of Pathology and [2]Department of Biomedical Science, Faculty of Medicine, University of Malaya, Kuala Lumpur, Malaysia and [3]Institute of Health and Community Medicine, University Malaysia Sarawak, Sarawak, Malaysia.

Send correspondence and reprint requests to:

Kum Thong Wong, MBBS, MPath, FRCPath, MD,

Department of Pathology, Faculty of Medicine,

University of Malaya, 50603 Kuala Lumpur, Malaysia;

E-mail: wongkt@ummc.edu.my

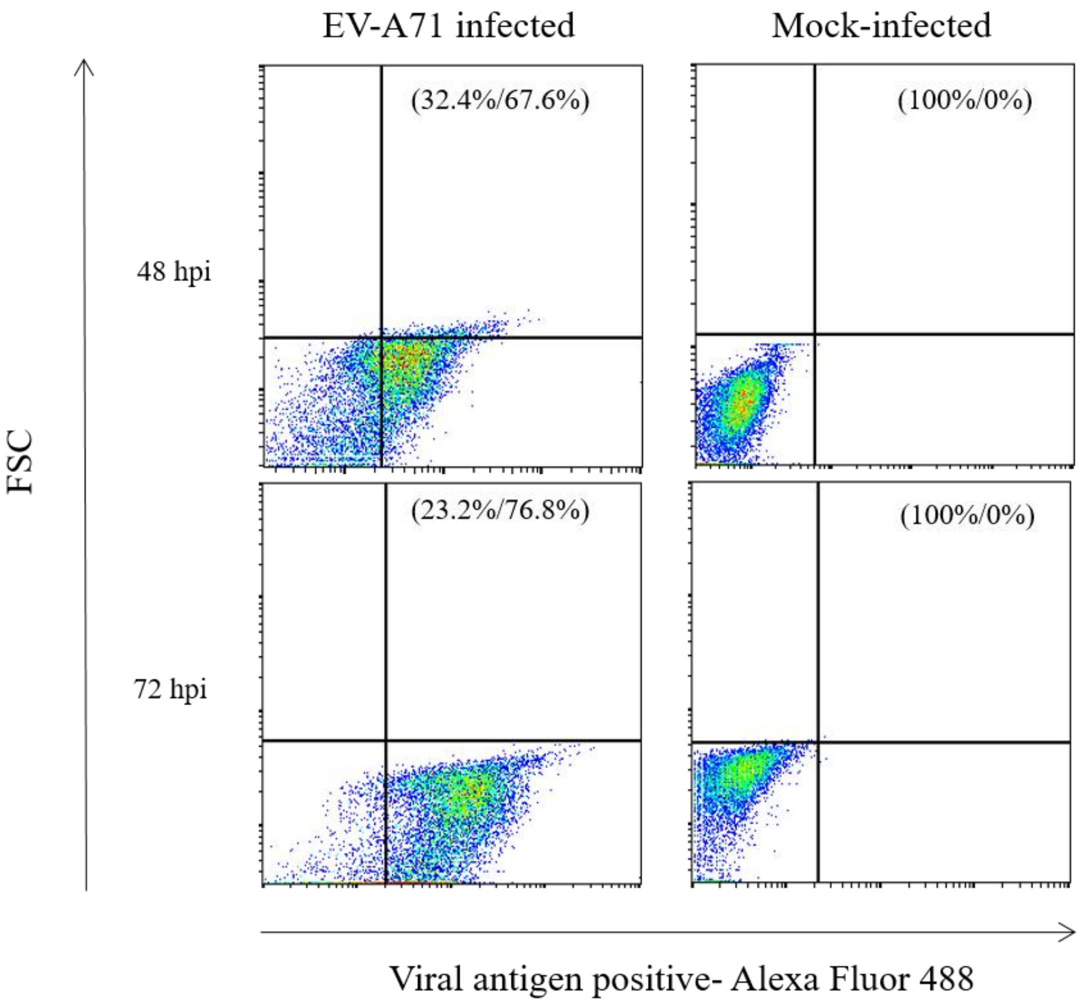


**Supplementary Figure S1 SK-N-SH cells infected at MOI of 10.** Flow cytometry analysis of SK-N-SH cells infected with EV-A71/13903 at 48 and 72 hours post-infection (hpi). Each graph is a representative set of similar triplicate results.

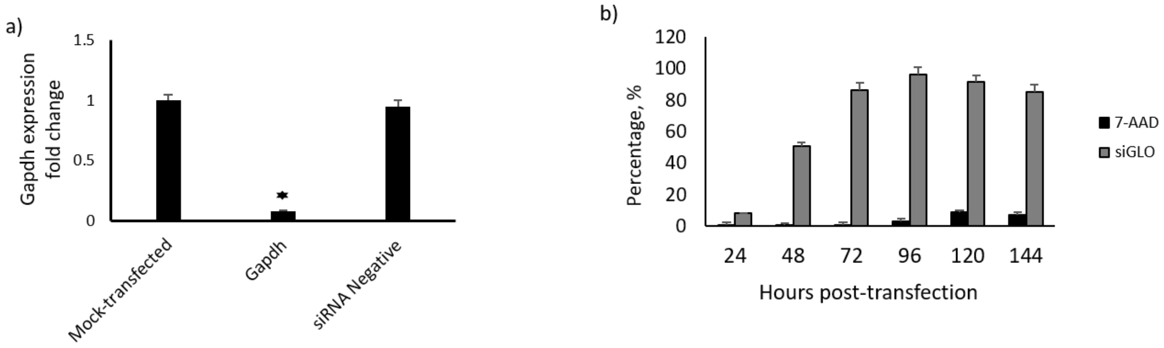


**Supplementary Figure S2 Transfection controls showed credible results.** (a) Gene expression of GAPDH in siRNA GAPDH and siRNA Negative transfected SK-N-SH cells at 48 hpt. GAPDH expression were significantly decreased (*$p<0.05$) in about 99% of SK-N-SH cells. siRNA Negative showed basal level expression of GAPDH. (b) Flow cytometry analysis of transfection efficiency and cell viability were determined using siGLO and 7-AAD, respectively. siGLO showed 86% cells to be successfully transfected at the 48 hpt, and 7-AAD staining showed only 1 – 8% of cell death at 24 to 144 hpt. All data represents the mean±standard deviation of a set of triplicates.

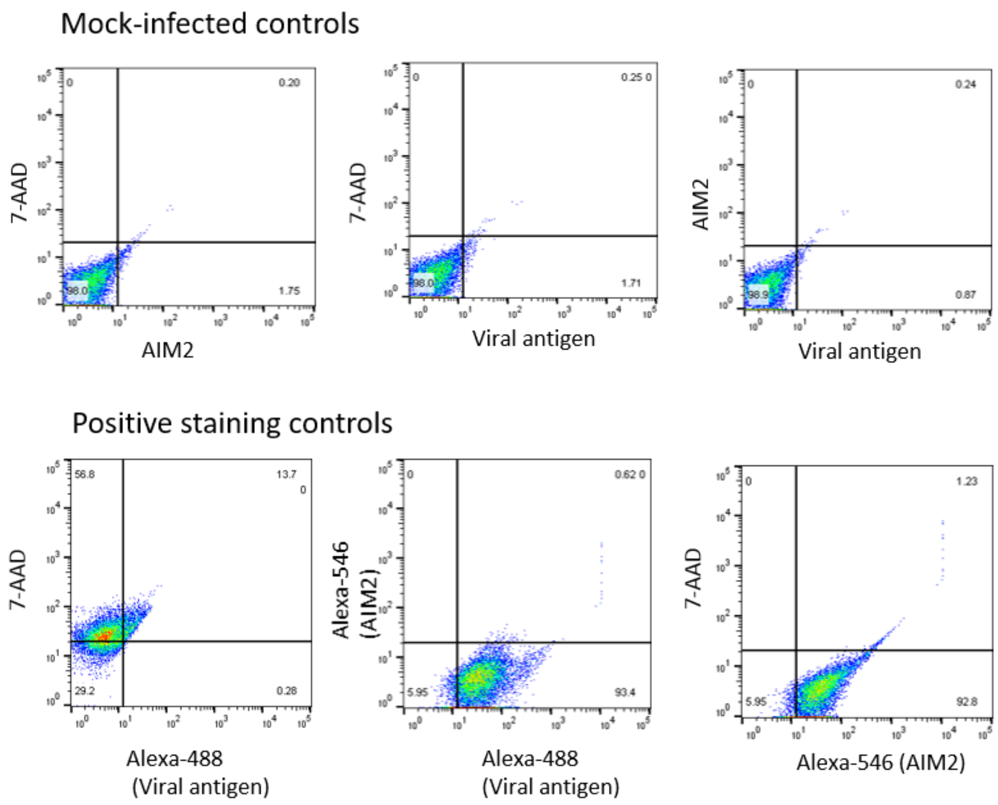


**Supplementary Figure S3 AIM2 and 7-AAD were not expressed in mock-infected SK-N-SH cells.** Flow cytometry of AIM2 expression, 7-AAD staining and viral antigens in SK-N-SH cells staining. Mock-infected SK-N-SH cells were negative for all stains. Positive controls for all staining was acceptable.

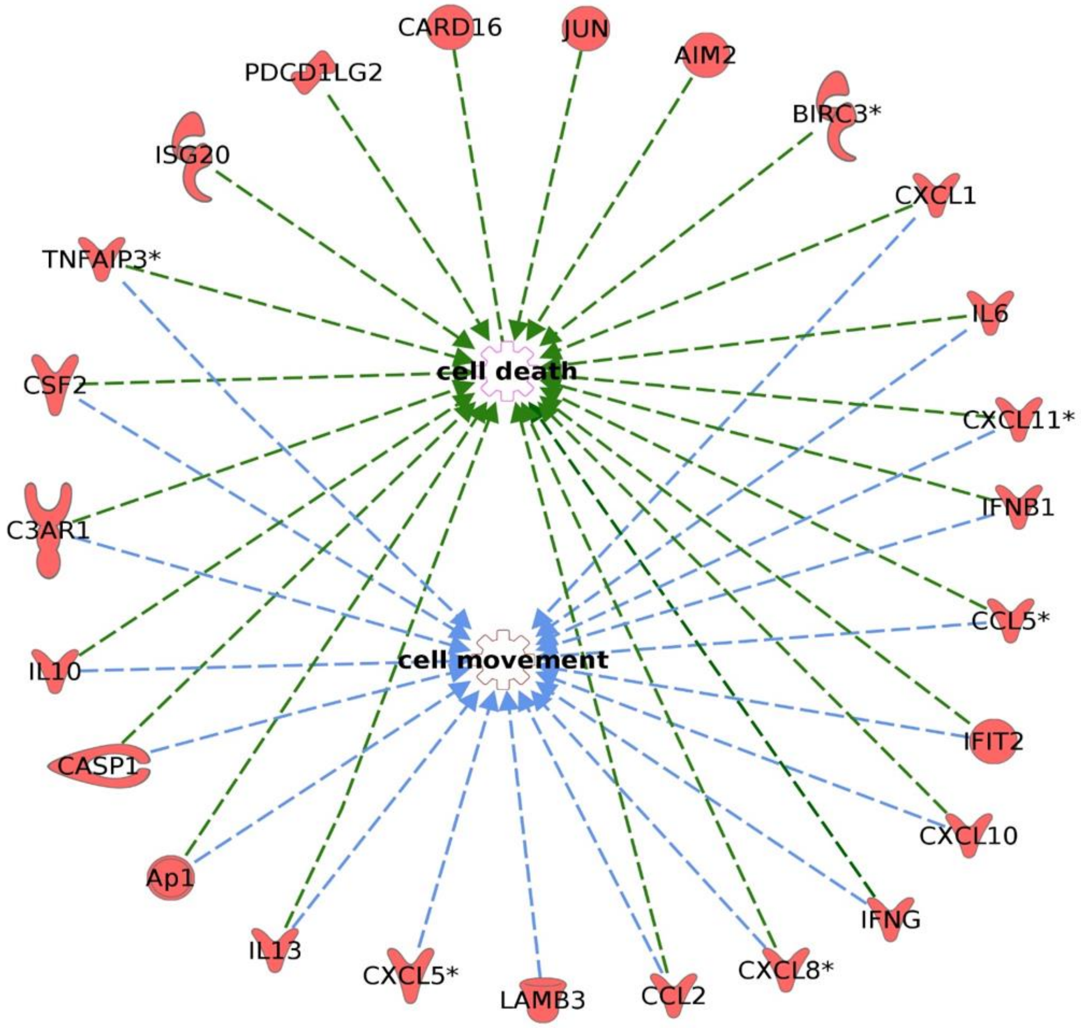


**Supplementary Figure S4 Disease and function pathways analysis indicates the overlap between cell death and cell movement pathways.** Most genes were involved in both cell death (green arrows) and cellular movement (blue arrows) mechanisms, including caspase-1 and inflammatory mediators, IL-6, CXCL10 and TNF-α. AIM2 and CARD16 is only involve in cell death mechanisms. All illustrated genes were up-regulated in SK-N-SH cells.
